# VEGF controls microglial phagocytic response to amyloid-β

**DOI:** 10.3389/fncel.2023.1264402

**Published:** 2023-12-15

**Authors:** Priscille de Gea, Sarah Benkeder, Pauline Bouvet, Mélanie Aimard, Naura Chounlamountri, Jérôme Honnorat, Le Duy Do, Claire Meissirel

**Affiliations:** ^1^Laboratory MeLIS, Institut Neuromyogène, Synaptopathies and Autoantibodies, INSERM U1314, CNRS UMR 5284, Université Claude Bernard Lyon 1, Lyon, France; ^2^French Reference Center for Paraneoplastic Neurological Syndromes and Autoimmune Encephalitis, Hospices Civils de Lyon, Hôpital Neurologique Pierre Wertheimer, Bron, France

**Keywords:** microglia, Alzheimer’s disease, VEGF, amyloid-β, TREM2, phagocytosis

## Abstract

Microglial cells are well known to be implicated in the pathogenesis of Alzheimer’s disease (AD), due to the impaired clearance of amyloid-β (Aβ) protein. In AD, Aβ accumulates in the brain parenchyma as soluble oligomers and protofibrils, and its aggregation process further give rise to amyloid plaques. Compelling evidence now indicate that Aβ oligomers (Aβo) are the most toxic forms responsible for neuronal and synaptic alterations. Recently, we showed that the Vascular Endothelial Growth Factor (VEGF) counteracts Aβo-induced synaptic alterations and that a peptide derived from VEGF is able to inhibit Aβ aggregation process. Moreover, VEGF has been reported to promote microglial chemotaxis to Aβ brain deposits. We therefore investigated whether VEGF could influence microglial phagocytic response to Aβ, using *in vitro* and *ex vivo* models of amyloid accumulation. We report here that VEGF increases Aβo phagocytosis by microglial cells and further characterized the molecular basis of the VEGF effect. VEGF is able to control α-secretase activity in microglial cells, resulting in the increased cleavage of the Triggering Receptor Expressed on Myeloid cells 2 (TREM2), a major microglial Aβ receptor. Consistently, the soluble form sTREM2 also increases Aβo phagocytosis by microglial cells. Taken together, these findings propose VEGF as a new regulator of Aβ clearance and suggest its potential role in rescuing compromised microglial function in AD.

## 1 Introduction

A key pathogenic mechanism in Alzheimer’s disease (AD) is the accumulation of the amyloid-β peptide (Aβ) in the brain, leading to its aggregation and deposition in plaques. Aβ plaque deposition occurs decades before patients are diagnosed with dementia, and is thought to result from an imbalance in the production and clearance of Aβ (Hardy and Selkoe, 2002; Mawuenyega et al., 2010). The neuropathological hallmarks associated with Aβ brain deposition are the dystrophic neurites surrounding dense-core plaques, along with activated microglial cells, forming the neuritic plaques (Serrano-Pozo et al., 2011). Dense-core Aβ plaques are constituted of compacted fibrillar Aβ surrounded by a halo of soluble Aβ oligomers or protofibrils, considered as the most neurotoxic Aβ forms (Koffie et al., 2009). Recent studies in mouse models of AD indicate that microglia play a pivotal role in the development of these plaques, either by contributing to their formation (Baik et al., 2016; Huang et al., 2021), or by limiting their expansion as a barrier to the addition of toxic soluble Aβ forms (Condello et al., 2015). Imaging studies using positron emission tomography markers have shown that brain Aβ load in patients with mild cognitive impairment (MCI) is associated with clusters of cortical microglial activation (Parbo et al., 2017). Furthermore, microglial activation at early MCI stages has been linked to higher gray matter and hippocampal volumes and slower decline in cognition, suggesting a protective role for activated microglia in early AD (Hamelin et al., 2016).

Late-onset AD (LOAD) which accounts for more than 90% of the cases implicate a combination of lifestyle, environmental and genetic factors, with a genetic contribution constituting 60 to 80% of the risk to develop the disease (Gatz et al., 2006). A major genetic risk factor for LOAD is the ε4 allele of the Apolipoprotein E gene *APOE* (Corder et al., 1993) that is associated with a decreased ability of the brain to clear accumulating Aβ in animal models of the disease (Castellano et al., 2011; Verghese et al., 2013). Most of the rare risk genes that have been identified by genome-wide association studies (GWAS) are strongly expressed in microglia, which emphasizes the role of these brain immune cells in AD pathogenesis (Jansen et al., 2019; Bellenguez et al., 2022). This includes a gene encoding for a key transmembrane receptor involved in microglial response, the Triggering Receptor Expressed on Myeloid cells 2 (TREM2), with variants increasing the risk for developing the disease (Guerreiro et al., 2013; Jonsson et al., 2013). Emerging evidence indicate that most TREM2 variants affect the interaction between TREM2 ligands and the receptor, resulting in either reduced or increased TREM2 activity *in vitro* (Gratuze et al., 2018). Finally, TREM2 activity can be impaired by its excessive shedding from the microglial cell surface, triggered by the α-secretases ADAM10 and 17 (a disintegrin and metalloproteinase domain-containing protein 10 and 17), in a specific AD-associated variant (Schlepckow et al., 2017; Thornton et al., 2017). The impact of TREM2 function has been investigated *in vivo* in AD mouse models deficient for TREM2 that show a clear reduction in Aβ plaque-associated microglia (Ulrich et al., 2014; Jay et al., 2015; Wang et al., 2015). In these models, TREM2 deletion also leads to an impairment in fibrillar Aβ deposition with loosely defined and less compact Aβ plaque morphology associated with dystrophic neurites (Wang et al., 2016), and at late stages, to an increase in Aβ load (Wang et al., 2015) and Aβ plaque size (Jay et al., 2017). Thus, TREM2 is implicated in microglial recruitment to Aβ plaques, in link with Aβ phagocytosis.

Phagocytosis carried out by microglia is important for tissue homeostasis in physiological conditions to clear the microenvironment from ineffective synapses, dying cells and cellular debris (Hammond et al., 2018). In AD, the role of microglia in Aβ plaque clearance is debated because pharmacological depletion of microglia in AD mouse models resulted in reduced Aβ plaque deposition when induced early and for a long duration period (Sosna et al., 2018; Spangenberg et al., 2019), but did not change Aβ load at later stages (Dagher et al., 2015; Spangenberg et al., 2016). Furthermore, when repopulation of microglia was initiated by discontinuous pharmacological treatments, it resulted in the remodeling of plaque morphology with plaque compaction and decreased neuritic dystrophy (Casali et al., 2020). However, with disease chronicity, microglia are thought to become hypofunctional and lose their ability to internalize and degrade Aβ aggregates (Friker et al., 2020). These studies underscore the complex role of microglia during the course of AD, being involved in initial plaque formation and preventing further neuronal damage by forming a barrier at the periphery. Thus, one still need to determine whether microglial internalization concern mostly freely diffusible neurotoxic Aβ forms or plaque-derived forms of fibrillar Aβ, and to explore the factors that can regulate this process in the plaque microenvironment.

Interestingly, the vascular endothelial growth factor (VEGF), which is known to display both a vascular and neuronal function (Lange et al., 2016), accumulates in Aβ plaques in the brains of AD patients and mouse models of the disease (Yang et al., 2004; Ryu et al., 2009; Martin et al., 2021). Evidence also show an impairment in VEGF protein expression in AD, with mainly an upregulation in the brain (Thomas et al., 2015; Miners et al., 2016), but contrasted results in the periphery, with up- or downregulation in the serum (Mateo et al., 2007; Huang et al., 2013; Cho et al., 2017; Liang et al., 2021). This increase in brain VEGF levels correlates with the amount of insoluble Aβ and with the progression of the disease (Thomas et al., 2015). The impact of VEGF has been further explored in mouse models of AD using transgenic mice overexpressing VEGF or with brain administration of VEGF-releasing nanospheres or secreting cells, and showed an improvement in memory deficits, in vascularization and a decreased Aβ load (Spuch et al., 2010; Herrán et al., 2013; Religa et al., 2013; Garcia et al., 2014). These findings demonstrating a beneficial role of VEGF in preclinical models of the disease led to the longitudinal exploration of VEGF levels changes with biomarkers of AD during human brain aging. Importantly, higher VEGF levels in cerebrospinal fluid (CSF) have been associated with increased brain metabolism, less hippocampal atrophy and cognitive decline over time in the presence of elevated AD biomarkers (Hohman et al., 2015; Tubi et al., 2021). Thus, although VEGF brain expression has been linked to Aβ accumulation, it is implicated in cognitive improvement and reduced Aβ load.

A still unresolved question is whether the neuroprotective role of VEGF which limits brain Aβ accumulation in AD models, involves the regulation of microglial phagocytic function in AD. Interestingly, VEGF has been shown to trigger the recruitment of microglia via their VEGFR1 receptor to Aβ-rich brain areas in adult rats administrated with Aβ (Ryu et al., 2009). These data suggest a role for VEGF signaling in microglial function in AD amyloid pathology. Therefore, to explore the role and underlying mechanisms of VEGF in microglial phagocytosis of Aβ species, we used various approaches including flow cytometry, biochemical and fluorescence-based substrate assays and cell imaging. Our findings indicate that VEGF signaling increases microglial phagocytic function for Aβ oligomers but not fibrils in various cellular and *ex vivo* AD models. Moreover, we describe how the ADAM protease activity induced by VEGF can be involved in TREM2-dependent regulation of microglial phagocytosis of Aβ oligomers.

## 2 Materials and methods

### 2.1 Animals

Postnatal (P1-P2) wild-type C57BL/6JRj female and male mice were used for primary mixed glial cell cultures. *Ex vivo* cryostat replenishment assay was performed on 14 month-old male APP/PS1-21 mice expressing a transgene combining human Amyloid-β Precursor Protein *APP*^KM670/671NL^ and presenilin 1 *L166P* mutations under the *Thy1* promoter (Radde et al., 2006). Genotyping was systematically carried out to validate the presence of the transgene. Intracardiac-perfusions were then performed with Dulbecco Phosphate buffer saline (DPBS) supplemented in Mg^2+^ and Ca^2+^ under deep isoflurane anesthesia. Brains were directly snap-frozen in isopentane and kept at −80°C prior to cryo-sectioning at 10 μm thick. Sections were then kept at −20°C until use. The study respected the European Community Council directive 2010/63/EU on the protection of animals used for experimental and scientific purposes, and housing followed the guidelines approved by the French Ethical Committee of the Lyon 1 University (DR2013-47).

### 2.2 Cell culture

All key resources and software are listed in Supplementary Table 1. Microglia murine N9 cell line was kindly provided by Christian Haass group (German Center for Neurodegenerative Diseases (DZNE), Munich). N9 cells were cultured in serum-supplemented medium including DMEM with L-glutamine, 10% fetal bovine serum and 1% penicillin-streptomycin (P-S), and used prior to 15 passages. Before treatment application, cells were serum-deprived overnight in DMEM, 2mM L-glutamine and 1% P-S. Cell seeding density per well was: for Western Blot 800,000 cells in 12-well plate; for peptidolytic assays 400,000 cells in 96-well plate; for flow cytometry assays 250,000 cells in 12-well plate; for ELISA assay 800,000 cells in 12-well plate.

For primary mixed cultures, newborn mice cortices and hippocampi were collected and cut into pieces in DPBS supplemented with 6 mg/mL glucose and 1% P-S. Tissue dissociation was performed by soft trituration, prior to a centrifugation step at 800 rpm, 4°C for 7 min to remove debris and myelin. Astrocytes and microglial cells were then plated on poly-L-ornithine (1.5 μg/ml) coated petri-dishes, and cultured for 2 weeks in serum-supplemented medium at 37°C with 5% CO_2_. Microglia were isolated and collected after 1 h of shaking at 90 rpm, 37°C, and centrifuged 20 min at 800 rpm, 4°C. Quantitative Iba1 immunostaining validated the high yield of microglia after isolation with 91.99 ± 4.38% of Iba1-positive cells. For cryostat replenishment assay, 250,000 primary microglia were plated per brain section, in microglial complete medium adapted from Bohlen et al. (2019) including DMEM/F12 with 1% P-S, 2 mM L-Glutamine, 5 μg/ml N-acetyl cysteine, 100 μg/mL apo-transferrin, 100 ng/mL sodium selenite, 1.5 μg/ml cholesterol, 0.1 μg/ml oleic acid, 1 ng/ml gondoic acid, 1 μg/ml heparan sulfate, 2 ng/ml TGFβ2 and 10 ng/ml M-CSF.

### 2.3 Western blotting

N9 cells were serum-deprived overnight and then treated with VEGF (50 ng/ml) for 5, 15 or 30 min. After a quick DPBS wash, cells were incubated 10 min in lysis buffer (25 mM Tris–HCl, 5 mM EDTA, 0.50% DOC, 150 mM NaCl, 1% NP-40, 0.10% SDS, pH 7), supplemented with protease inhibitor and phosphatase inhibitor cocktails and 2 mM orthovanadate. Cell lysates were next collected and kept 10 min on ice prior to a centrifugation step at 10,000 *g*, 4°C for 10 min. Supernatants were kept at −20°C until use for immunoblotting. Denatured samples (15 μg of proteins) were separated using 4–12% SDS-PAGE gels, transferred on nitrocellulose membranes and immunoblotted after a blocking step in Tris-buffered saline, 0.1% Tween (TBS-T) and 5% milk. Primary antibodies were incubated overnight at 4°C in 2% milk TBS-T, prior to an incubation step with appropriate secondary HRP-conjugated antibodies for 1.5 h at room temperature (RT). Proteins were visualized with an ECL-containing solution and signal intensity quantified with ImageJ software. To compare the signaling pathways activated by VEGF in N9 microglia, we have assessed the phosphorylated and total protein expressions on the same membrane after stripping, and proper loading was verified using β-actin. Then, the ratio between the phosphorylated and total protein expression per condition, or between the phosphorylated form and the housekeeping β-actin was calculated.

### 2.4 ADAM peptide substrate assay

ADAM10/17 cell surface activity was measured by monitoring the cleavage of the fluorogenic peptide substrate III (Mca-PLAQAV-Dpa-RSSSR-NH2). For short treatments, N9 microglial cells were directly seeded in HBSS to avoid interference of the medium with real-time monitoring of peptide fluorescence. VEGF (50 ng/ml) was applied simultaneously or 15 to 30 min prior to fluorogenic substrate (5 μM) addition. Fluorescence kinetics was then monitored from 10 min after substrate addition for 90 min using a TECAN microplate reader, at 320 nm of excitation and 390 nm of emission. Each time point was normalized to the total fluorescence of the control and expressed as Relative Fluorescence Unit (RFU) plotted as a function of time to calculate slopes (RFU/min/%Control). For longer treatments ranging from 2 to 20 h, N9 cells were seeded overnight in serum-free medium for adhesion, and VEGF (50 ng/ml) was applied in serum-free medium for 2, 6 or 20 h followed by a complete medium change to HBSS and fluorogenic substrate (5 μM) addition. Fluorescence level was then measured 30 min after substrate addition and RFU values were normalized to control. In both assays, DAPI (50 μg/ml) was incubated for 30 min at the end of the experiment and fluorescence determined at 360 nm of excitation and 465 nm of emission to correct substrate fluorescence values as a function of cell density.

### 2.5 *In vitro* phagocytosis assay

FAM-Aβ was reconstituted in 5% DMSO-DPBS, sonicated for 20 min in ice-cold bath and centrifuged for 3 min at 10,000 rpm 4°C to remove non-reconstituted material. Theoretical FAM-Aβ concentration was considered as 92 μM and aliquots were stored at −20°C until use. Aβ oligomers (Aβo) and fibrils (Aβf) were obtained after, respectively 2 or 72 h of aggregation of a 15 μM equivalent monomer solution at 37°C. VEGF (50 ng/ml) and recombinant sTREM2 (20 nM) treatments were systematically applied 5 min prior to 1 μM Aβo or Aβf incubation.

In all phagocytic experiments using flow cytometry, membrane-bound Aβ fluorescent signal was discriminated from internalized Aβ signal by incubating N9 microglial cells at 4°C to inhibit phagocytosis (Aβ-4°C condition). For Aβo phagocytosis, N9 cells were collected from 0 to 8 h after Aβo application. For Aβo degradation experiment, N9 cells were first incubated 1 h with Aβo to induce phagocytosis prior to complete medium removal, and Aβ degradation was monitored after 4 or 8 h. For Aβf phagocytosis, cells were collected 4 h after Aβf incubation.

Cells were collected after a 5 min incubation in 0.25% Trypsin-DPBS. Washing steps and incubation solutions were performed in DPBS-Mg^2+/^Ca^2+^ with 0.5% BSA. For Aβo phagocytosis assay, cells were first stained for 30 min in Ghost Dye Violet 450 for cell viability. Non-specific binding to N9 Fc-receptors was blocked by a 5 min incubation step with CD16/32 antibody prior to labeling with CD11b-PE or control isotype (IgG2b-PE) for 15 min. In the other phagocytosis and degradation assays, only Ghost-Violet labeling was performed to validate microglial viability. Cells were next fixed in 1% paraformaldehyde, washed and analyzed by flow cytometry using BD FACSCanto II™ Flow Cytometer with BD FACSDiva and FlowJo software.

The Aβ-4°C condition allowed defining gates for intracellular (Int) or total (Membrane + Intracellular) Aβ on FITC histograms. For Aβo phagocytosis and degradation assays, the total Aβ Mean Fluorescence Intensity (MFI) obtained after VEGF treatment was normalized to control 0 h. For phagocytosis assays with Aβf and VEGF as well as Aβo and sTREM2, the internalized Aβ MFI was normalized to control 0 h.

### 2.6 *Ex vivo* phagocytosis assay

*Ex vivo* cryostat replenishment assay was adapted from previous studies (Bard et al., 2000; Claes et al., 2019; Colombo et al., 2021). Briefly, wells were built around thawed APP/PS1 brain cryostat sections in sterile conditions and washed once with DPBS. On the first day *in vitro* (DIV1), Aβ antibody (6E10) was incubated for 1 h on consecutive sections prior to seeding (cells) or not (no cells) with 250,000 primary microglial cells in complete medium, and cultures were maintained at 37°C and 5% CO2. Control or VEGF (50 ng/ml) treatments were applied on DIV2 and DIV4 with half medium change. Sections were fixed on DIV5 with 4% paraformaldehyde for 10 min.

For immunostainings, a permeabilization and blocking step was performed in PBS - 0.1% Triton - 5% BSA for 45 min and sections were incubated overnight at 4°C with primary anti-Aβ (4G8) and anti-Iba1 antibodies in PBS-0.3% Triton-1% BSA. Secondary anti-mouse-Alexa 555 or anti-rabbit-Alexa 488 antibodies were next applied for 2 h at RT. Finally, DAPI counterstaining was performed for 10 min and sections were mounted in Fluoromount-G medium with coverslips. Whole slide images were acquired using the Axio-Scan.Z1 slide scanner and 3D-high resolution acquisitions were performed with Confocal Zeiss 880 (CIQLE platform, Lyon). Images were further analyzed with MATLAB^®^2018A by selecting Aβ positive plaques with a minimum area of 50 μm^2^.

### 2.7 ELISA sTREM2

N9 cell culture media were collected after 1 to 4 h of VEGF (50 ng/ml) treatment, centrifuged 5 min at 500 *g*, and supernatants were kept at −20°C until use. 96-well microplates were coated overnight at 4°C with 0.5 μg/ml of TREM2 antibody in PBS. After a blocking step of 1 h with DPBS-3%BSA, sTREM2 standard (0–100 pg/ml) or samples were loaded in wells and incubated 2 h, at RT. Biotinylated-TREM2 antibody (1 μg/ml) was then used as a detection antibody and incubated for 2 h prior to HRP-conjugated streptavidin for 1 h, as reported (Zhong et al., 2019). Finally, reaction was performed with Substrate TMB reagent for 20 min and stopped with sulfuric acid solution. Absorbance was measured with a TECAN Microplate-reader successively at 450 nm and 540 nm.

### 2.8 Statistical analysis

Data were expressed as mean ± SEM, except for Figure 3C where median and quartiles box plot were shown. All data and statistics are available in Supplementary Tables 2, 3. For western blot, flow cytometry, peptidolytic and ELISA assays, sample size (n) refers to the number of culture wells or petri-dishes and N refers to the number of experiments. For *ex vivo* assay, n refers to the number of brain sections and N refers to the number of mice. Data representation and analyses were carried out with RStudio software. Descriptive statistics and appropriate tests were systematically used to evaluate normality and homoscedasticity of data, and to determine the appropriate statistical analysis to be carried out using non-parametric or parametric tests. Details of each statistical analysis between treatments or groups using ANOVA, Wilcoxon or Kruskal-Wallis are presented in figure legends. For phagocytosis, replenishment and ELISA assays, we used mixed effect models adapted to complex experimental designs with dependent and non-dependent data, and allowing the inclusion of random factors in addition to fixed effects. Model fitting and analysis were carried out with the lme4 package (Bates et al., 2015). For *in vitro* phagocytosis experiments, condition (control/VEGF) and time were considered as fixed effects, whereas the experiment factor was considered as a random intercept allowing us to improve model fitting in comparison with classical linear model. For *ex vivo* phagocytosis, fixed effects were the interaction between replenishment and condition, with a random intercept for the sections. For sTREM2 ELISA assay, the condition was a fixed effect, with a random intercept for the experiment factor. *p < 0.05; **p < 0.01 and ***p < 0.001.

## 3 Results

### 3.1 VEGF signaling induces Src family kinase activation

**FIGURE 1:**
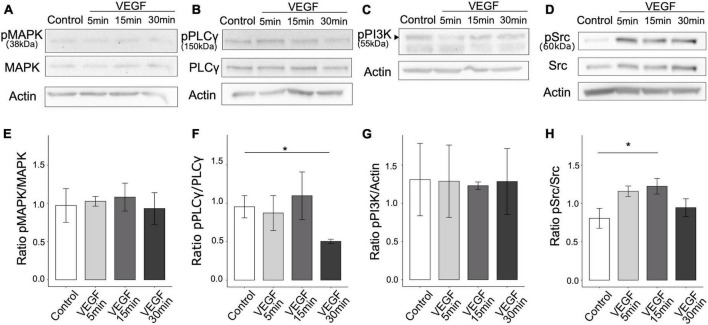
VEGF activates SFK pathway in microglial cells. N9 microglial cells were treated with 50 ng/ml of VEGF (gray shades) for 5, 15, or 30 minutes (min), or not treated (control, white), prior to cell lysis and western blotting analysis of signaling pathway activation. **(A–D)** Representative immunoblots of N9 cell lysates using antibodies against specific phosphorylated residues or total proteins for MAPK-p38 pathway (phosphoMAPK T180/182, MAPK-p38 total), PLCγ (phosphoPLCγ Y783, total PLCγ) and Src pathway (phosphoSrc Y416 and total Src). Note that the expression level of the phosphorylated form of p55 regulatory subunit of PI3K, indicated by a black arrowhead (phosphoPI3K Y199 residue), was compared to the housekeeping protein β-actin **(C)**. β-actin expression validated the proper loading of each sample **(A–D)**. **(E–H)** Expression ratios between phosphorylated and total proteins **(E,F,H)** or actin **(G)** show that VEGF increased SFK activation [**(H)**, one-way ANOVA, *p* = 0.04886, Dunnett *post hoc* *p < 0.01, *n* = 5], but did not change MAPK-p38 activation [**(E)**, Kruskal-Wallis, *p* = 0.7271, *n* = 5] nor PI3K-p55 regulatory subunit phosphorylation [**(G)**, Kruskal-Wallis: *p* = 0.9529, *n* = 5]. PLCγ activation was decreased after 30 min of VEGF stimulation [**(F)**, Kruskal-Wallis: *p* = 0.0447, Dunn *post hoc* with Holm’s correction, *p < 0.05, *n* = 5].

To determine whether VEGF is involved in the regulation of microglial clearance function, we first assessed the presence of the main VEGF receptors, VEGFR1 and VEGFR2, on N9 microglial cells and primary microglia. We confirmed using western blotting that both N9 cells and primary microglia only express VEGFR1 but not VEGFR2 (Supplementary Figure 1A). Next, we explored the signaling pathways downstream of VEGFR1 based on those previously identified in endothelial cells (Koch et al., 2011), focusing on phospholipase C-γ (PLCγ), mitogen-activated protein kinase (MAPK-p38), phosphatidylinositol-3 kinase (PI3K-p55) and Src. We quantified signaling protein expression changes following VEGF treatment of N9 microglia for 5, 15, or 30 minutes by targeting specific phosphorylated residues on immunoblots. The ratio of phosphorylated over total protein expression level was considered as an indicator of pathway activation (Figures 1A–D). VEGF treatment didn’t induce changes in MAPK-p38 (T180/182), nor in PI3K-p55 (Y199) activation, but triggered a late reduction in PLCg (Y783) activation (Figures 1E–G). In contrast, Src family kinase (SFK) phosphorylation at residue Y416 was significantly increased after 15 minutes of VEGF treatment compared to control (Figure 1H). This VEGF-induced SFK activation was transient. Thus, these findings indicate that VEGF initially promotes microglial activation of SFK pathway, likely via VEGFR1.

### 3.2 VEGF promotes microglial phagocytosis of Aβ oligomers

As SFK activation is involved in microglial phagocytosis (Portugal et al., 2022) and microglial phagocytosis required to limit Aβ accumulation in the brain in AD (Hellwig et al., 2015), we investigated whether VEGF affects Aβ uptake by microglia. Firstly, as soluble Aβ oligomers (Aβo) are considered the most toxic Aβ species in AD (Hong et al., 2018), we examined the kinetics of Aβo-mediated phagocytosis by N9 microglia using biotinylated Aβo aggregated for 2 h. Immunofluorescent analyses were performed to monitor the intracellular localization of Aβo versus surface adhesion by comparing Aβo-treated N9 cells to endogenous biotin signal in non-treated cells. We observed streptavidin labeling in microglia as soon as 2 h after oligomer treatment, which remained at a stable and high intensity level up to at least 8 h, in contrast to the control group (Supplementary Figure 2A). To further characterize Aβo internalization process in N9 microglia, we assessed the lysosomal packaging of fluorescent Aβo (FAM-Aβo) after 4 h of treatment. The partial colocalization of FAM-Aβo with the microglial lysosomal marker CD68 confirmed N9 microglial phagocytic activity and Aβo uptake in phagolysosome compartments (Supplementary Figure 2B). Altogether, these results indicate that Aβo internalization by N9 cells is saturable in a time-dependent manner and involves a phagocytic process.

**FIGURE 2:**
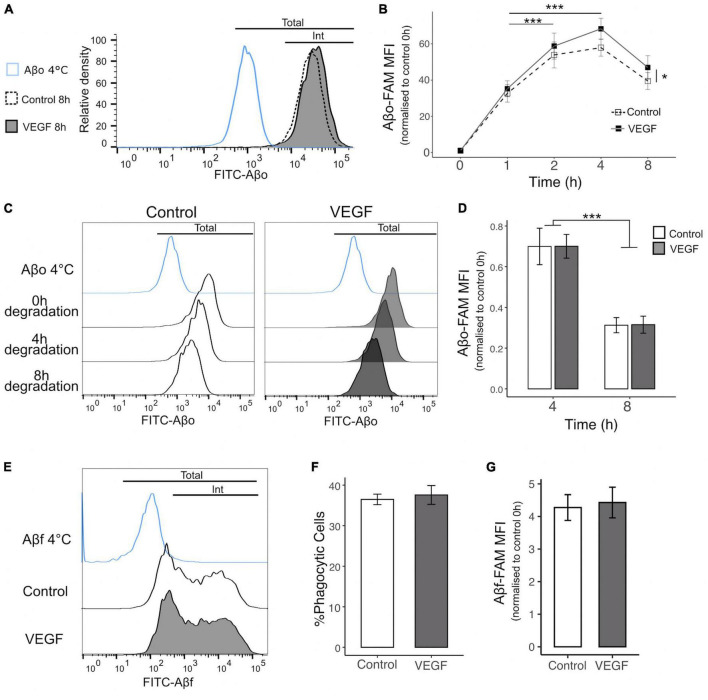
VEGF increases microglial phagocytosis of Aβo but not Aβf. The phagocytic uptake of FAM-Aβo or FAM-Aβf was measured in microglial N9 cells pre-treated or not with VEGF (50 ng/ml) for 5 minutes prior to FAM-Aβ application, and quantified by flow cytometry analysis. **(A)** Representative histogram of FAM-Aβo FITC measurement in N9 cells in control condition (white, dashed line) or with VEGF (gray) treatment, after 8 h of phagocytosis. The Aβ-4°C condition (blue) showed a moderate cell surface binding of Aβo in absence of phagocytosis and allowed to determine the FITC threshold for internalized (Int) vs. total Aβo amount (cell surface and internalized). **(B)** Changes in FITC fluorescence over time expressed as the normalized Mean Fluorescence Intensity (MFI) to 0 h (h) illustrating kinetics of FAM-Aβo phagocytosis in control condition (white, dashed) or with VEGF (black) treatment. Control and VEGF conditions showed a time-dependent shift toward higher MFI values. In addition, VEGF induced a significant increase in MFI values compared to control, reflecting an increase in Aβo phagocytosis (linear mixed-effect model; significant difference between the conditions, Control-VEGF *p < 0.05; time-dependent effect for both conditions between 1 h–2 h and 1 h–4 h ***p < 0.001, *n* = 8). **(C)** Changes in FITC fluorescence over time showing FAM-Aβo degradation for control (left, white) or VEGF (right, gray) treatment after 1 h of internalization and complete removal of the medium. Intracellular Aβ degradation was followed for 4 and 8 h. **(D)** Quantitative analysis showing a significant reduction of normalized FAM-Aβo MFI after 8 h compared to 4 h in control and VEGF-treated conditions, indicating time-dependent degradation of internalized FAM-Aβo. VEGF did not change the degradation kinetics compared to control (two-way ANOVA, Control-VEGF n.s; time ***p < 0.001, *n* = 4). **(E)** Representative histograms displaying FAM-Aβf FITC measurements after 4 h of phagocytosis in control (white) or VEGF (gray) treated condition. **(F)** Percentage of FAM-Aβf phagocytic microglia with or without VEGF treatment (Wilcoxon test n.s, *n* = 6). **(G)** Quantification of internalized FAM-Aβf MFI, with or without VEGF treatment (Wilcoxon test n.s, *n* = 6).

Next, we determined the impact of VEGF on Aβo internalization process using flow cytometry to quantify FAM-Aβo microglial uptake overtime, with or without VEGF treatment (Figures 2A, B). The phagocytic uptake of FAM-Aβo by N9 microglia cells was characterized by a green fluorescent peak clearly shifted toward higher fluorescence intensity values than in the control condition performed at 4°C, a control group in which phagocytosis is blocked (Figure 2A). When comparing the relative fluorescence normalized to the initial time, a rapid increase in fluorescence occurred during the first 2 h, followed by a slower increase up to 4 h and a moderate decrease from 4 to 8 h, which may reflect a balance between phagocytosis and degradation. Importantly, VEGF significantly increased the mean fluorescence intensity over time and compared with the control condition, indicating that microglial cells were capable of taking higher amount of Aβo when treated with VEGF (Figures 2A, B). To determine if this VEGF effect was due to an increase in Aβo phagocytosis or alternatively to an impairment in intracellular Aβ degradation, we performed additional degradation studies. We therefore examined if the VEGF would change the intracellular Aβ content overtime following a 1-h incubation step of N9 microglia with Aβo, during which microglia have previously been shown to phagocyte Aβo (Figure 2B). Intracellular Aβ degradation was monitored after 4 and 8 h by measuring the relative fluorescence intensity per cell (FAM-Aβ-MFI) normalized to 100% after the internalization step (0 h degradation) (Figures 2C, D). Microglia efficiently degraded internalized Aβo, with approximately 30% and 70% reduction for, respectively 4 and 8 h of degradation (Figure 2D). Notably, the VEGF did not alter the progressive decline in microglial Aβ content compared to the control condition. Altogether, our findings demonstrate that VEGF increases microglial Aβo uptake, without modifying intracellular degradation capacity.

### 3.3 VEGF does not change microglial phagocytic ability for Aβ fibrils

Various Aβ species are recognized by specific microglial receptors and induce distinct phagocytic responses depending on their conformation, namely the oligomeric (Aβo) versus the fibrillar (Aβf) forms (Yu and Ye, 2015). To further characterize VEGF functional effect in N9 microglial phagocytosis, we explored its impact on Aβf uptake by monitoring the internalization of fluorescent FAM-Aβf, aggregated for 72 h (Figures 2E–G). Based on the bimodal distribution of Aβf fluorescence, we can define two distinct microglial populations, the one with a low fluorescence intensity per cell and a second population with higher phagocytic ability characterized by a broader peak (Figure 2E). Importantly, neither the proportion of phagocytic cells nor the amount of intracellular Aβf differed between control and VEGF treated cells (Figures 2F, G). From this assay we concluded that N9 microglia can display various phagocytic mechanisms of Aβf, but VEGF plays no role in this process.

**FIGURE 3:**
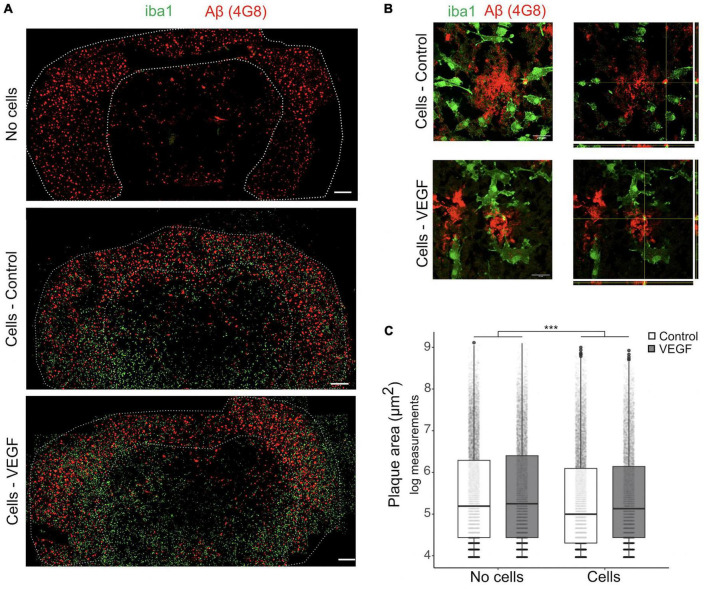
VEGF doesn’t change amyloid plaque clearance *ex vivo*. **(A)** Representative immunofluorescent staining of APP/PS1 brain sections treated with or without VEGF and replenished (cells) or not (no cells) with primary microglial cells. Mouse microglial cells were cultured on unfixed cryostat brain sections from 14-month-old APP/PS1 mice, with or without VEGF treatment (50 ng/ml) for 4 days. Sections were fixed and immunostained for microglia (Iba1, green) and Aβ-plaques (4G8 antibody, red), and white dashed-lines delineate the cortex. Scale bar, 500 μm. **(B)** 3D confocal images of control or VEGF replenished APP/PS1 brain sections showing that primary microglia are localized around Aβ-plaques (z-stack, left panels), with processes in the same focal plane (right panel, orthogonal view). Yellow puncta depict Aβ internalized by microglia, as indicated by crossing white lines in the orthogonal view. Scale bar, 20 μm. **(C)** Quantitative comparison of cortical amyloid plaque area in control or VEGF-treated APP/PS1 brain sections, replenished or not with microglia. Data were transformed with natural logarithm and each dot corresponds to a plaque with boxplot representing median and quartiles (linear mixed-effect model, cells ***p < 2.10^–16^, condition (Control/VEGF) n.s, condition × cells n.s, *n* = 5 slices).

As the response of microglial cells to brain Aβf deposition in plaque is a hallmark of AD, we next used an *ex vivo* plaque clearance assay (Bard et al., 2000; Xiang et al., 2016; Colombo et al., 2021) to further investigate VEGF impact on microglia Aβf phagocytosis. Cryostat brain sections from APP/PS1 mice were used as an extracellular matrix on which primary microglia were cultured to assess their Aβ plaque clearance capacity. In this AD model, amyloid plaques accumulate in the cortex from 8 weeks onward (Radde et al., 2006), with plaque load stabilization occurring at 10 months (Yan et al., 2009). Primary murine microglia were therefore deposited on 14-month-old APP/PS1 brain sections and cultured with or without treatment for 5 days to allow assessment of plaque reduction. First, we validated that microglia presence on brain sections reduces plaque size and that their distribution heterogeneity did not bias our measurements (Supplementary Figure 3). Based on Aβ immunostaining, plaque area was measured in the cortex with a cut-off value of 50 μm^2^ and compared between consecutive slices, replenished or not with microglia (Figure 3A). Further 3D confocal analyses were performed to document intracellular uptake of Aβ by microglia around plaques, using orthogonal view (Figure 3B). Our results show that replenishment of APP/PS1 brain sections with primary microglia significantly reduced Aβ plaque size, but without any difference between control and VEGF conditions (Figure 3C). Taken together, these findings indicate that VEGF doesn’t promote synthetic and natural Aβ fibrils or plaque phagocytosis by microglia. This suggests that VEGF could be involved in the regulation of microglial molecular mechanisms that mediate soluble Aβ oligomer but not fibrillar Aβ internalization.

### 3.4 VEGF induces ADAM10/17 protease activity in microglia

**FIGURE 4:**
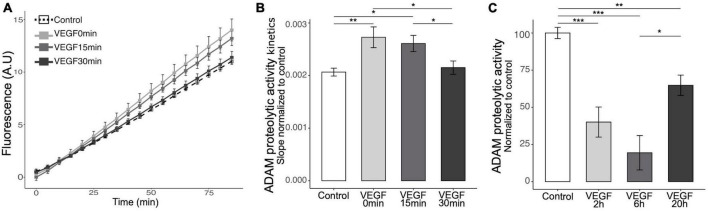
Acute VEGF treatment increases microglial cell surface protease activity of ADAM10 and ADAM17. **(A)** Kinetics of cell surface protease activity of microglial ADAM10 and ADAM17 expressed as Relative Fluorescence Unit (RFU) per minute (min) was assessed by incubating control or VEGF-treated N9 microglia with a soluble fluorogenic peptide substrate. N9 cells were pre-treated or not with VEGF (50 ng/ml) for 15 or 30 min prior to peptide substrate addition, or with concomitant application, and VEGF treatment was maintained during the experiment. Fluorescence of cleaved substrate was monitored for 90 minutes at 320 nm excitation and 390 nm emission. **(B)** Quantitative comparison from normalized slope kinetics curves shown in panel **(A)** indicates that VEGF triggers a time-dependent increase in ADAM proteolytic activity, only significant for acute VEGF pre-treatments (Kruskal-Wallis *p* = 0.0014, Dunn *post hoc* with Holm’s correction, *p < 0.05 and **p < 0.01, *n* = 15). **(C)** Quantitative analysis shows that longer VEGF pre-treatment duration, ranging from 2 to 6 and 20 h (h), induces a significant reduction in cell surface protease activity of ADAM10/17, maximal after 6 h (Kruskal-Wallis *p* = 6.33 × 10^–7^, Dunn *post hoc* with Holm’s correction, *p < 0.05, **p < 0.01 and ***p < 0.001, *n* = 15).

Microglia can bind soluble Aβ oligomers through cell surface receptors that trigger a phagocytic response and receptor shedding is critical for regulating this process (Dhandapani et al., 2022). To explore the mechanisms underlying VEGF effect on microglia, we focused on the possibility that it modulates the activity of the major sheddase in microglia, namely ADAM10 and ADAM17 (Lambrecht et al., 2018; Hsia et al., 2019). Previous studies reported that VEGF is able to increase ADAM10 and ADAM17 expression and activity *in vitro* in endothelial cells and *in vivo* in the brain of an AD mouse model (Donners et al., 2010; Guo et al., 2019). We therefore investigated if VEGF modulates these proteases activity specifically in microglia, and have first confirmed that N9 microglial cells and primary microglia expressed ADAM10 and ADAM17 (Supplementary Figures 1B, C). Next, we evaluated the activity of the proteases using a fluorogenic peptide substrate whose sequence is specifically recognized by ADAM10 and ADAM17. Once cleaved, the substrate emits a fluorescence that can be reported to the ADAM peptidolytic activity. Short VEGF pre-treatment or concomitant application of VEGF and substrate significantly increased the ADAM10/17 peptidolytic activity compared to control (Figures 4A, B). In contrast, a longer pre-treatment failed to stimulate ADAM10/17 cleavage activity, suggesting a rapid and transient effect of VEGF. To further test the kinetics of VEGF action on ADAM10/17 activity, we treated N9 cells with VEGF for longer durations and evaluated the proteases cleavage ability. Interestingly, these VEGF treatments exhibited an inhibitory effect on ADAM10/17 activity compared to control (Figure 4C). Taken together, these findings revealed a time-dependent effect of VEGF on ADAM10/17 protease activity, suggesting that this activity is transiently upregulated at the microglial cell surface, then sustainably downregulated by a VEGF-dependent process.

### 3.5 VEGF induces microglial TREM2 shedding and sTREM2 release

**FIGURE 5:**
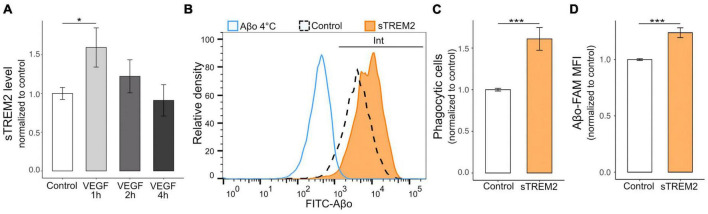
VEGF promotes microglial TREM2 shedding with sTREM2 release, and sTREM2 increases Aβo phagocytosis. **(A)** Microglial TREM2 ectodomain shedding was assessed by measuring sTREM2 level released in the culture medium by N9 cells in control condition (white) or treated with 50 ng/ml VEGF (gray) for 1 to 4 h (h). Quantitative comparison using an ELISA assay revealed that short VEGF treatment for 1 h increased TREM2 shedding and sTREM2 release (linear mixed-effect model, control-VEGF 1 h *p < 0.05, *n* = 7). **(B)** Representative histogram of FAM-Aβo FITC measurements in N9 cells in control condition (white, dashed line) or pre-treated with 20 nM of sTREM2 (orange) for 5 minutes prior to FAM-Aβo application for 4 h. The Aβ-4°C condition (blue) allowed to determine the FITC threshold for internalized (Int) Aβo. Note the clear shift in FITC fluorescence in sTREM2-treated cells reflecting an increase in FAM-Aβo uptake. **(C)** Proportion of Aβo phagocytic microglia normalized to control, with or without sTREM2 treatment, showing that sTREM2 stimulates N9 cell phagocytosis (Wilcoxon test, ***p < 0.001, *n* = 8). **(D)** Quantification of internalized FAM-Aβf MFI normalized to control shows that sTREM2 treatment induces Aβf-FAM uptake (Wilcoxon test, ***p < 0.001, *n* = 8).

ADAM10/17 perform two types of cleavage depending on the target, including the peptidolytic cleavage of soluble substrates and the shedding of transmembrane proteins at the cell surface (Reiss and Bhakdi, 2017). To determine the relevance of the VEGF effect on ADAM10/17 sheddase activity, we evaluated the extent of ectodomain release of a microglial Aβ receptor targeted by these proteases, TREM2 (Kleinberger et al., 2014; Feuerbach et al., 2017). We first validated TREM2 expression on N9 microglial cells and primary microglia using western blotting (Supplementary Figure 1D), confirming a previous report (Xiang et al., 2016). N9 microglia were then treated with VEGF for 1 to 4 h, and the levels of the soluble form of TREM2 (sTREM2) were determined by ELISA assay on collected culture medium (Figure 5A). The results demonstrated that VEGF significantly increases sTREM2 levels only after 1 h of treatment, which is consistent with the early VEGF-dependent increase in ADAM10/17 protease activity shown previously.

As the functional relevance of sTREM2 is not fully understood (Brown and St George-Hyslop, 2021), we designed experiments to determine whether sTREM2 in only a byproduct of TREM2 cleavage or whether it could promote Aβo phagocytosis by microglia. Recombinant sTREM2 was therefore administered to N9 microglia 5 minutes prior to FAM-Aβo, and intracellular Aβo fluorescence was measured after 4 h, when Aβo phagocytosis is at its peak (Figure 5B). Importantly, we observed that sTREM2 significantly increased both the number of phagocytic cells, i.e., the proportion of cells that have internalized Aβo, and the amount of internalized Aβo (Figures 5C, D). Collectively, these findings indicate that VEGF transiently stimulates ADAM10/17 protease activity in microglial cells resulting in TREM2 shedding with sTREM2 release. In turn, sTREM2 was shown to promote Aβo phagocytosis by microglial cells and could be considered as a mediator of VEGF functional effect on Aβ uptake.

## 4 Discussion

The present study demonstrates that VEGF promotes microglial phagocytosis of Aβ oligomers but does not affect fibrillar Aβ internalization. Our experimental data show that this enhanced phagocytosis of Aβo is closely linked to the time-dependent effect of VEGF on ADAM10/17 proteolytic activity. VEGF, by transiently activating ADAM metalloproteases, is implicated in TREM2 shedding and sTREM2 production, and therefore regulates TREM2 phagocytic function.

Increasing evidence demonstrate that VEGF exerts its effects upon binding to membrane tyrosine kinase receptors in endothelial cells (Simons et al., 2016), but the type of receptor and molecular pathways involved in VEGF regulation of microglia function are largely unknown. Our present study now shows that SFK signaling is activated upon VEGF treatment in VEGFR1-expressing microglial cells. Although VEGFR1 is commonly considered as a decoy receptor due to its low tyrosine kinase activity (Shibuya, 2006), the genetic invalidation of its tyrosine kinase domain in mice has been shown to impair macrophage migration, implying a direct VEGFR1 signaling (Hiratsuka et al., 1998). Moreover, recent findings indicate that VEGFR1 plays an active signaling role in the development of retinal microglial cells (Ogura et al., 2017) and in the regulation of microglial receptors involved in phagocytosis (Xu et al., 2017). In AD pathological context, VEGFR1 has been shown to underlie microglial recruitment to brain Aβ deposits in response to VEGF (Ryu et al., 2009). Such a chemotactic response of microglia to VEGF may play a key role in the regulation of Aβ phagocytosis, because we and others have previously shown that VEGF accumulates in and around Aβ plaques in the brain of AD patients (Yang et al., 2004; Ryu et al., 2009; Thomas et al., 2015; Martin et al., 2021).

We therefore investigated whether VEGF may serve as a priming signal to regulate Aβ phagocytosis by microglia, and examined its impact on Aβ uptake by taking in account different Aβ conformations with soluble or fibrillar Aβ species. Notably, our findings revealed that VEGF promotes an efficient uptake of soluble Aβo overtime, without stimulating fibrillar Aβ phagocytosis. Furthermore, we confirmed that VEGF does not impair intracellular Aβo degradation, demonstrating that this VEGF-dependent increase in intracellular Aβo results solely from an increased phagocytosis by microglia. Importantly, the kinetics of Aβo uptake upon VEGF treatment reach a plateau after 4 h of treatment, indicating that the underlying mechanisms is saturable and may likely involve a receptor-mediated phagocytic process rather than macropinocytosis (Mandrekar et al., 2009).

Brain Aβ deposition in plaques is thought to result from an increasing Aβ accumulation that leads to the formation of aggregates due to an impaired clearance process (Tarasoff-Conway et al., 2015). To determine if the VEGF facilitating effect on Aβo phagocytosis plays a role in the microglial clearance of preexisting plaques, we used an *ex vivo* assay, as previously reported (Bard et al., 2000; Xiang et al., 2016; Colombo et al., 2021). In the presence of antibody stimulation, VEGF does not impact the microglial-mediated amyloid plaque clearance, in agreement with our observation on VEGF-independent fibrillar phagocytosis. Antibody-mediated microglial phagocytosis has been shown to involve Fcγ-receptors (FcγR) (Bard et al., 2000; Wilcock et al., 2004; Doens and Fernández, 2014) and some of these receptors, such as the FcγRIIb, can interact with Aβo (Kam et al., 2013; Amin and Harris, 2021). Therefore, the question remains to determine why there is no difference with VEGF treatment in oligomeric Aβ deposited around plaques, while it promotes Aβo phagocytosis by microglia. We envisioned that VEGF may regulate a FcγR-independent pathway in microglia, focusing on TREM2 for two main reasons. First, TREM2 deficiency in microglia impairs cell migration toward a defined chemoattractant *in vitro* (Mazaheri et al., 2017), mimicking the impact of VEGFR1 blockade on microglia (Ryu et al., 2009). Second, TREM2 is able to directly bind Aβo with a higher affinity (Zhao et al., 2018) than that of Aβ fibrils (Lessard et al., 2018).

One of the main mechanisms regulating TREM2 function in microglial phagocytosis is the receptor ectodomain shedding by ADAMs, giving rise to soluble TREM2 (sTREM2) (Feuerbach et al., 2017; Schlepckow et al., 2017, 2020). Notably, VEGF has previously been reported to increase ADAM10 expression and activity in endothelial cells, where ADAM10 interacts with VEGFR2 triggering an increase in endothelial cell permeability and migration (Donners et al., 2010). Moreover, VEGF gain of function has been shown to enhance brain ADAM10 expression in the Tg2576 mouse model of AD, alleviating Aβ load and cognitive deficits (Guo et al., 2019). Importantly, our findings demonstrate that VEGF transiently increases ADAM10/17 proteolytic activity in microglial cells, which solely express VEGFR1 but not VEGFR2. The mechanism underlying this VEGF-induced activation of ADAM10/17 metalloproteases could involve the Src-Family kinase (SFK) pathway, as we already showed that VEGF triggers SFK activation in microglial cells. Indeed, a crosstalk has been identified between ADAM10 and Src kinases, implicating Src activation as a positive regulator of ADAM10 shedding activity in pituitary adenoma cells (Huang et al., 2020). However, our findings also revealed that the short-term effect of VEGF, triggering an increase in ADAM10/17 proteolytic activity, is different from the changes induced by long-term VEGF exposure. The downregulation of ADAM10/17 activity at the microglial cell surface after long-term VEGF stimulation could be due to their internalization and/or ectodomain shedding by ADAM9 (Tousseyn et al., 2009), also activated by VEGF (Mehta et al., 2018).

In agreement with the time-dependent increase of ADAM10/17 proteolytic activity by VEGF, we uncovered an enhanced production of sTREM2 which is released in the culture medium after 1 h, and normalized after 2 h. In contrast, the subsequent blockade of ADAM10/17 proteolytic activity due to VEGF could stabilize at later stages the TREM2 receptor on the microglial cell surface. Emerging evidence now indicate that sTREM2 exerts direct functions and is not merely a degradation by-product that serves as a decoy receptor to trap TREM2 ligands (Yang et al., 2020; Brown and St George-Hyslop, 2021; Filipello et al., 2022). However, the role of sTREM2 in AD pathogenesis is still debated. On the one hand, sTREM2 production by receptor shedding is thought to negatively regulate TREM2 function, as the AD associated H157Y TREM2 variant promotes this shedding, leading to reduced TREM2-dependent phagocytosis (Schlepckow et al., 2017; Thornton et al., 2017). Furthermore, the inhibition of TREM2 shedding using an antibody directed against an epitope close to the ADAM10/17 cleavage site, stabilizes TREM2 on the cell surface, activates its signaling pathway, and leads to Aβ phagocytosis by microglia (Schlepckow et al., 2020). On the other hand, when the ADAM10/17 cleavage site is genetically substituted in mice to reduce TREM2 cleavage, the stabilization of membrane-bound TREM2 facilitates Aβ deposition and neuronal dystrophy in an AD mouse model (Dhandapani et al., 2022). Also, a gain of function of sTREM2 in 5xFAD mice by viral gene transfer or brain injection revealed its protective role in facilitating Aβ phagocytosis, plaque clearance, and in preventing memory deficits (Zhong et al., 2019).

Given these conflicting reports, we therefore investigated whether sTREM2 could directly impact Aβo phagocytosis in our microglial cell model. Our findings showed that sTREM2 treatment promotes microglial phagocytosis of Aβ even when administrated at a much lower level than previously reported (Zhong et al., 2019), where non-physiological levels were applied compared to human CSF (Suárez-Calvet et al., 2019). The key question is now to understand how the VEGF-induced release of microglial sTREM2 could underlie the associated increase in Aβo phagocytosis. The high affinity binding of TREM2 ectodomain for Aβo and the slow dissociation kinetics of the complex (Lessard et al., 2018) could be instrumental in the blockade of Aβ aggregation, as it has been recently shown for a VEGF-derived peptide (Kober et al., 2020; Vilalta et al., 2021; Bouvet et al., 2023). Thus, the time-dependent effect of VEGF on microglial ADAM10/17 proteolytic activity may result in an early blockade of Aβ aggregation via sTREM2 release, followed by a subsequent TREM2-mediated increase in the phagocytosis of small oligomeric species.

Taken together our findings provide evidence that VEGF is able to promote the clearance of toxic Aβ species by stimulating microglial phagocytosis. This VEGF-mediated response could be hindered in APP/PS1 mice and AD patients’ brain because VEGF has been shown to accumulate in insoluble Aβ plaques where it might be trapped (Yang et al., 2004; Ryu et al., 2009; Thomas et al., 2015; Martin et al., 2021). Such a trapping could lead to a depletion of free VEGF available to microglial cells, resulting in reduced uptake of Aβ oligomers in the course of AD. Thus, we propose a model in which VEGF signaling pathway in microglia could influence their ability to clear Aβ oligomers and contain plaque extension in early stages of AD.

## Data availability statement

The original contributions presented in this study are included in this article/Supplementary material, further inquiries can be directed to the corresponding author.

## Ethics statement

The study respected the European Community Council directive 2010/63/EU on the protection of animals used for experimental and scientific purposes, and housing followed the guidelines approved by the French Ethical Committee of the Lyon 1 University (DR2013-47). The study was conducted in accordance with the local legislation and institutional requirements.

## Author contributions

PG: Conceptualization, Writing—original draft, Formal analysis, Investigation, Methodology, Writing—review and editing. SB: Formal analysis, Methodology. PB: Methodology, Investigation, Writing—review and editing. MA: Investigation, Methodology. NC: Investigation, Methodology. JH: Funding acquisition, Resources. LD: Formal analysis, Investigation, Methodology. CM: Conceptualization, Funding acquisition, Supervision, Writing—original draft, Writing—review and editing.
