# Supplementary material for: VEGF controls microglial phagocytic response to amyloid-β
Source: Front Cell Neurosci. 2023 Dec 15;17:1264402. doi: 10.3389/fncel.2023.1264402 (PMC10757340; doi:10.3389/fncel.2023.1264402)
Supplement: Supplementary file 1 [file Data_Sheet_1.pdf]

Supplementary Material

1 Supplementary Materials and Methods

1.1 Western blotting of membrane protein expression

Primary and N9 microglia were seeded in serum-supplemented medium and HUVEC (human umbilical vein endothelial cells) were cultured in wells coated with collagen I, in a medium composed of MV2 heparin-free and 1% P-S. After a quick DPBS wash, cells were incubated 10 minutes with lysis buffer (25 mM Tris-HCl, 5 mM EDTA, 0.50% DOC, 150 mM NaCl, 1% NP-40, 0.10% SDS, pH 7), supplemented with 2% protease inhibitor and 2% phosphatase inhibitor cocktails with 2% orthovanadate, and 0.02% Benzonase was also added for HUVEC. Cells were then scraped and collected, lysates were kept 10 minutes on ice prior to a centrifugation step at 10,000 g, 4°C for 10 minutes. Supernatants were kept at -20°C until use.

Denatured samples (20 µg of proteins for microglia, 10µg for HUVEC) were separated using 4-12% SDS-PAGE gels, and then transferred on nitrocellulose membranes. Membrane saturation, antibody incubation and revelation were similar to the protocol described in the main material section. The primary antibodies used here are: VEGFR1 (1/2000, Abcam 32152, RRID:AB_778798), VEGFR2 (1/1000, Cell Signaling Technology-2479, RRID:AB_2212507), ADAM10 (1/2000, Abcam 1997, RRID:AB_302747), ADAM17 (1/2000, Sigma SAB3500367, RRID:AB_10643826), TREM2 (1/500, ThermoFisher AF1729, RRID:AB_354956) and β-Actin (1/10000, Sigma-Aldrich A1978, RRID:AB_476692). Antibodies were revealed with goat anti-rabbit HRP-conjugated antibody (1/10000, Jackson ImmunoResearch 111-036-003, RRID:AB_2337942), donkey anti-sheep (1/5000, ThermoFisher-A16041, RRID:AB_2534715) HRP-conjugated antibody or goat anti-mouse (1/10000, Jackson ImmunoResearch 115-036-003, RRID:AB_2338518) HRP-conjugated antibody.

1.2 Phagocytosis immunofluorescence assay

Phagocytosis assay was performed to determine ability and kinetics of Aβ uptake by N9 microglia. Biotinylated-Aβ (1-42) was reconstituted as previously described (Martin et al., 2021) and aggregated at 15 µM for 2 hours at 37°C to form a mix of oligomers. N9 cells were plated in serum-free medium (60,000 cells/cm^2) and treated with 1 µM of biotinylated-Aβ for 0 to 8 hours or 1 µM of FAM-Aβ for 4 hours of phagocytosis. Cells were then quickly washed with DPBS and fixed

with 4% paraformaldehyde in 0.1 M phosphate buffer at RT for 15 minutes. For immunostaining, a permeabilizing step was performed in PBS-0.3%Triton-1%BSA and anti-iba1 (1/500, Wako 19741, RRID:AB_839504) or anti-CD68 (1/1000, Bio-Rad MCA1957, RRID:AB_322219) antibody was incubated overnight. Secondary anti-rabbit-Alexa 647 antibody (1/300, ThermoFisher A21244, RRID:AB_2535812) or anti-rat-Alexa 647 (1/300, ThermoFisher A21247, RRID:AB_141778) was applied, together or not with streptavidin-488 (1/500, ThermoFisher S11223) and phalloidin-Alexa 555 (1/500, ThermoFisher 34055), prior to DAPI (1 µg/ml) counterstaining. 3D Images were obtained under identical acquisition parameters with a Zeiss-880 confocal microscope equipped with a 63X objective and deconvolved with Huygens Professional software (CIQLE platform, Lyon), prior to 2D maximum intensity projection. For lysosomal packaging analysis, individual N9 microglial cells were defined as a Region of interest (ROI) based on a permissive CD68 threshold of 10%. In each ROI, CD68 and FAM-Aβ fluorescent signals were defined as positive signals with a fivefold more restrictive threshold of 2 %. Binary masks were then extracted for CD68 and FAM-Aβ positive signals and the spatial colocalization of these two signals was assessed and expressed as a percentage of intracellular FAM-Aβ or CD68 per ROI for each coverslip.

1.3 Validation of the *ex vivo* phagocytosis assay

Prior to the quantitative plaque analysis shown in Figure 3, we first validated (1) the impact of primary microglia on plaque phagocytosis and (2) the influence of cell seeding and distribution variability on brain sections. All the analyses were performed only on the cortex.

The detection of Aβ plaque fluorescence intensity, number and area (>50 μm^2) was expressed as a ratio to the overall cortical area, allowing to illustrate the data as Aβ intensity, relative Aβ area (Area Aβ/Total Area) and mean plaque area.

To check if microglia heterogenous distribution on section could influence the data, a regional analysis was carried out on control slices only. Cortical region of interest (ROI) encompassing a 600 µm-square were randomly selected and Aβ plaques as well as microglial surface were measured. Cortical ROIs placed at the same location between replenished and non-replenished sections were used to calculate the change in relative Aβ area. Three categories of microglial coverage (in μm^2) reflecting microglial density were defined per ROI: (1) 1000 to 30,000 μm^2, (2) 30,000 to 50,000 μm^2 and (3) more than 50,000 μm^2. The relative Aβ area in each ROI was expressed as a function of cell density. A mixed model analysis was used with density categories as fixed effect and a random intercept for slice section and for ROI.

2 Supplementary Figures

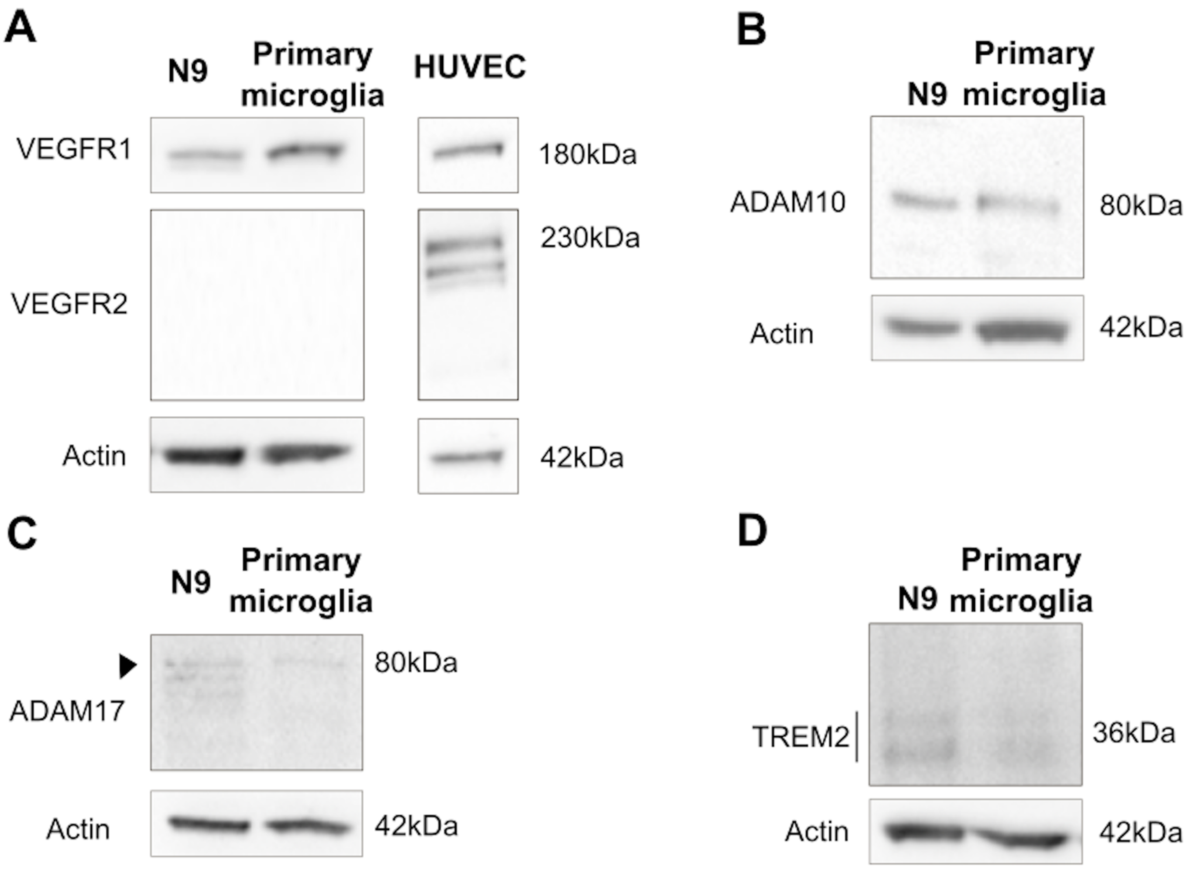

Supplemental Figure 1 – N9 and primary microglia express VEGFR1, ADAM10, ADAM17 and TREM2

(A) Representative immunoblot from N9 microglia, primary microglia and HUVEC lysates showing that microglia express only VEGFR1 and not VEGFR2, while endothelial cells express both receptors. **(B-D)** Immunoblots of N9 and primary microglia lysates illustrating ADAM10 (B), ADAM17 (C) and TREM2 (D) expression, with related β-actin expression for loading control (n=4, N=2).

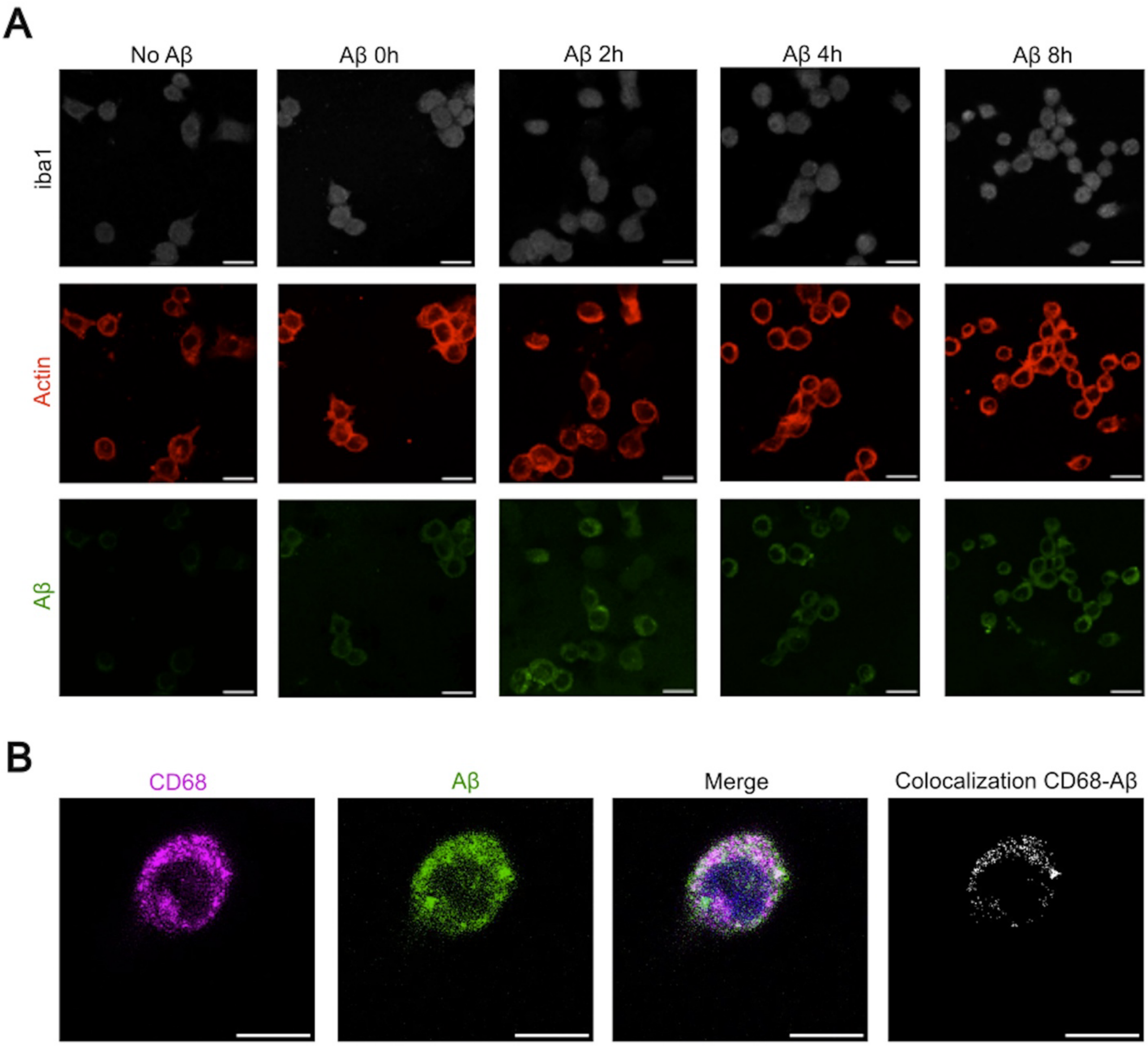

Supplemental Figure 2 – Aβ uptake by N9 microglia

(A) Representative maximum projections of 3D confocal images of N9 microglia immunostainings showing biotinylated Aβ phagocytosis over time, up to 8 hours. Intracellular microglial compartment was identified with Iba1 (white) and Phalloidin-Actin (red) combined staining and validated Aβ internalization (green). Scale bar, 20 µm. **(B)** Representative maximum projections of 3D confocal images of a N9 microglial cell showing CD68 immuno-positive lysosomes (magenta), FAM-Aβ (green) and merge composite with additional DAPI counterstaining, after 4 hours of phagocytosis. A threshold was applied to CD68 and Aβ fluorescent signals, and their colocalization was assessed using a binary image reflecting lysosomal FAM-Aβ. Lysosomal FAM-Aβ was expressed as a percentage of total intracellular Aβ with 31.84 ± 1.15 % of total Aβ contained in lysosomes (N=3 n=8 coverslips, 30 cells analyzed per coverslip). Scale bar, 10 µm.

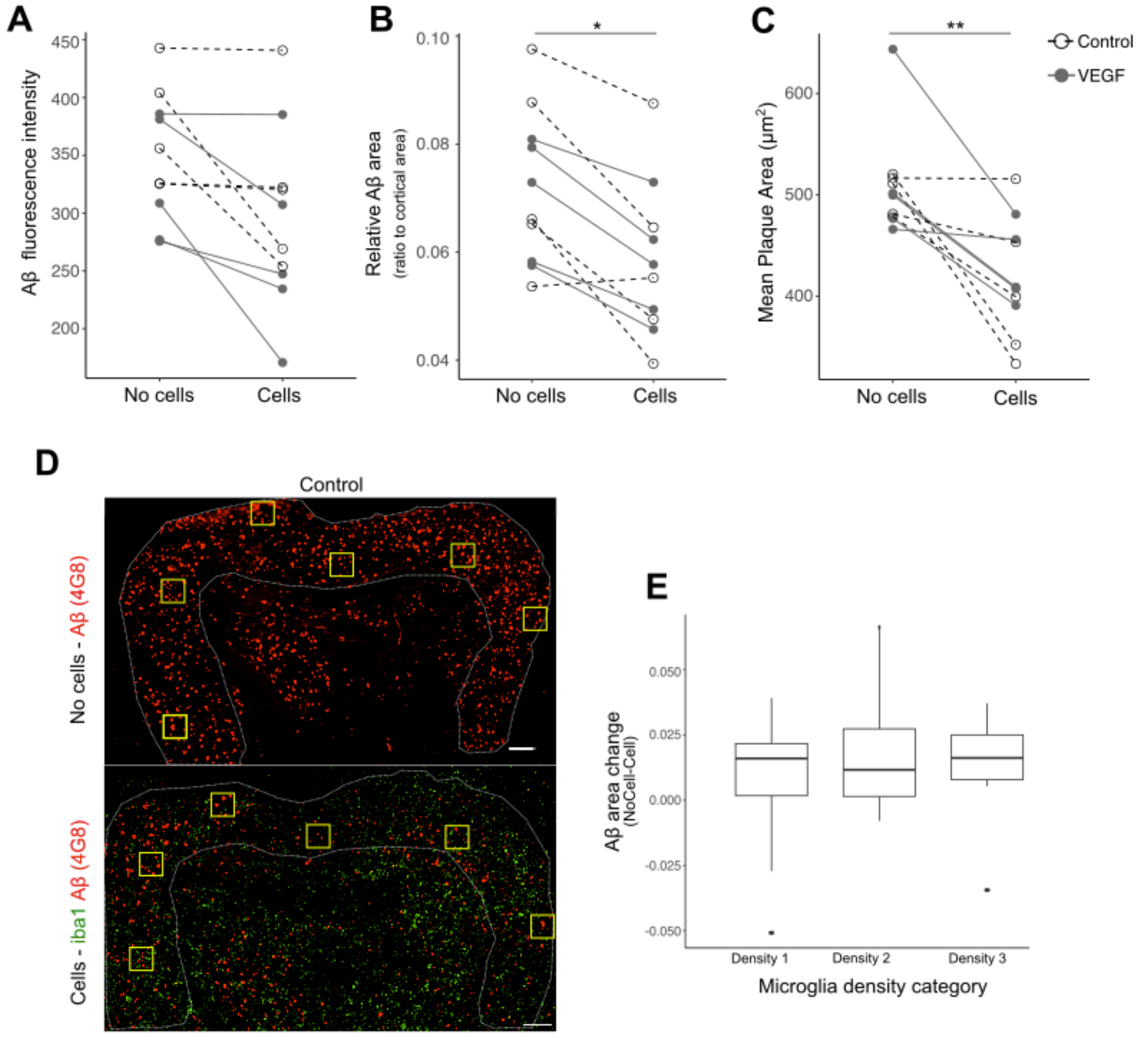

Supplemental Figure 3 - Validation of the *ex vivo* model of Aβ plaque clearance
(A-C) Quantitative analysis of total cortical Aβ immunostaining in brain sections with or without primary microglia replenishment. Each point represents a brain section in control (dashed-lines) or VEGF (50 ng/ml, solid lines) treated condition. **(A)** Aβ mean fluorescence intensity did not significantly changed in presence of primary microglial cells (Wilcoxon test, $p=0.05243$, $n=5$). **(B)** Note that the ratio of Aβ immunostaining area to the total cortical area is decreased in the presence of microglial cells (Wilcoxon test, $*p=0.0433$, $n=5$). **(C)** The mean Aβ plaque size is also decreased in the presence of cells (Wilcoxon test, $**p=0.0015$, $n=5$). **(D)** Region of interest (ROI) analysis used to assess the impact of microglial cell density on Aβ plaque size. Representative confocal images of Aβ (4G8, red) and microglia (Iba1, green) immunostainings with ROI delineated by yellow squares. Cortical ROI were analysed at the same location on two consecutive brain sections, with or without primary microglia seeding, and microglial density as well as Aβ immunostaining areas were measured per ROI. **(E)** Quantitative comparison of Aβ area changes between No cell and Cell ROI according to microglial density category. Cell density doesn't impact Aβ plaque size (linear mixed-effect model, density category, $p=0.6314$) (41 ROI, $n=5$ slices).

3 Supplementary Tables

3.1 Table caption

Supplementary Table 1: List of models, material, products and software used in the study.
Supplementary Table 2: Data summary, for main and supplemental figures.
Supplementary Table 3: Statistics summary, for main and supplemental figures.

3.2 Supplementary Tables

Supplementary Table 1

Reagent or Resource	*Source*	*Reference*
**Antibodies/Probes**		
Rabbit anti-phospho Src Tyr416 WB: 1/1000	Cell Signaling Technology	CST2101 RRID: AB_331697
Rabbit anti-Src WB: 1/1000	Cell Signaling Technology	CST2108 RRID: AB_331137
Rabbit anti-phospho PI3K p85(Tyr458)/p55(Tyr199) WB: 1/1000	Cell Signaling Technology	CST4228 RRID: AB_659940
Rabbit anti-PI3K p85 WB: 1/1000	Cell Signaling Technology	CST4257 RRID: AB_659889
Rabbit anti-phospho MAPK p38 (Thr180/Thr182) WB: 1/1000	Cell Signaling Technology	CST9211 RRID: AB_331641
Mouse anti-MAPK p38 WB: 1/1000	Abcam	ab31828 RRID: AB_881839
Rabbit anti-phospho PLCɣ (Tyr783) WB: 1/1000	Cell Signaling Technology	CST2821 RRID: AB_330855
Rabbit anti-PLCɣ WB: 1/1000	Cell Signaling Technology	CST2822 RRID:AB_2163702
Mouse anti-β Actin WB: 1/40000	Sigma-Aldrich	A1978 RRID: AB_476692
Goat anti-rabbit HRP WB: 1/10000	Jackson Immunoresearch	111-036-003 RRID:AB_2337942
Goat anti-mouse HRP WB: 1/10000	Jackson Immunoresearch	115-036-003 RRID:AB_2338518
Rat anti-CD11b-PE FC: 20µg/ml	BDBioscience	557397 RRID: AB_396680
Mouse control isotype PE FC: 20µg/ml	BDBioscience	555058 RRID: AB_395678
Rat anti-CD16/36 Fc Block FC: 1µg/ml	BDBioscience	553142 RRID: AB_394657
Ghost Dye Violet 450 FC: 0.25µl/tube	Cell Signalling Technology	49826
Mouse anti-Aβ 6E10 Cryostat: 5µg/ml	BioLegend	803002 RRID:AB_2564654
Mouse anti-Aβ 4G8 Cryostat IF: 1/500	BioLegend	800709 RRID:AB_2565325
Rabbit anti-iba1 Cryostat IF: 1/500	Wako	19741 RRID:AB_839504

Goat anti-rabbit Alexa488<br>Cryostat IF: 1/500	ThermoFisher Scientific	A11034<br>RRID:AB_2576217
Goat anti-mouse Alexa 555 Cryostat<br>IF: 1/1000	ThermoFisher Scientific	A21424<br>RRID:AB_141780
DAPI 1µg/ml (IF), 50 µg/ml (Fluorogenic)	SigmaAldrich	D9542
Sheep anti-TREM2 antibody<br>ELISA: 0.5 µg/ml	R&D Systems	AF1729<br>RRID:AB_354956
Biotinylated sheep anti-TREM2 antibody<br>ELISA:1 µg/ml	R&D Systems	BAF1729<br>RRID:AB_356109
**Biochemistry products**		
Sodium orthovanadate	SigmaAldrich	450243
Phosphatase inhibitor cocktail	MERCK Milllipore	Cat#524629
cOmplete protease inhibitor cocktail	MERCK Millipore	Cat# 04693159001
Bolt MOPS SDS Running Buffer	Thermofisher Scientific	2550875
4-12% Criterion XT Bis-Tris Protein Gel	Bio-Rad	Cat#3450124
Nitrocellulose membrane 0.2 µm	Dutscher	10600001
SuperSignal West Pico Plus<br>Chemiluminescent Substrate	ThermoFisher Scientific	Cat#34577
Corning® 96 Well Black Polystyrene<br>Microplate	SigmaAldrich	CLS3603
Fluoromount G	Thermofisher Scientific	00-4958-02
Epredia SuperFrost Plus glass slides	Thermofisher Scientific	J1800AMNZ
Reagent Diluent Concentrate 2	R&D Systems	Cat#DY995
Clear polystyrene microplates	R&D Systems	Cat#DY990
Streptavidin HRP	R&D Systems	Cat#DY998
Substrate Reagent Buffer	R&D Systems	Cat#DY999B
Stop solution 2N Sulfuric acid	R&D Systems	Cat#DY994

Reagent or Resource	*Use*	*Source*	*Reference*
**Cell culture**			
DMEM Low glucose Phenol red	N9 microglia, Mixed culture	ThermoFisher Scientific	
DMEM Low glucose no Phenol red	N9 microglia	ThermoFisher Scientific	31885023
DMEM/F12 no Phenol Red	Primary microglia	ThermoFisher Scientific	
Fetal Bovine Serum	N9 microglia, Mixed culture	Eurobio	CVFSVF00 01
Penicillin-Streptomycin		ThermoFisher Scientific	11548876
L-Glutamine		ThermoFisher Scientific	25030024
N-Acetyl Cysteine	Primary microglia	Merck	A9165
Sodium Selenite	Primary microglia	Merck	AS5261

Apo-Transferrin	Primary microglia	Sigma-Aldrich	T1147
Recombinant murine M-CSF	Primary microglia	PreProtech	315-02
Heparan sulfate	Primary microglia	Amsbio	GAG-HS01
Oleic acid	Primary microglia	Merck	O1383
Gondoic acid	Primary microglia	Merck	E3635
Cholesterol	Primary microglia	Merck	700000P
Trypsin	N9 microglia	ThermoFisher Scientific	11538876
Dulbecco Phosphate Buffer Saline (DPBS)	N9 microglia	ThermoFisher Scientific	12037539
DPBS Mg^{2+} Ca^{2+}	Perfusion, FC	ThermoFisher Scientific	11580456
Hank's Balanced Saline Solution (HBSS)		Sigma	H6648
**Chemical, peptides, recombinant protein**			
Recombinant human VEGF 165		R&D System	293-VE
FAM-Amyloid-β protein (1-42)	FC	Bachem	4090151
Recombinant mouse TREM2-His	ELISA, FC	Interchim	50149-M08H
Fluorogenic substrate III (Mca-PLAQAV-Dpa-RSSSR-NH2)		BioTechne	ES003

**Experimental models**	
N9 microglia cell line	Christian Haass lab
APP/PS1-21 transgenic mice	Radde et al.,2006
C57Bl/6JRj	JanvierLab
**Software**	
ImageLab	Version 6.0.1
Fiji - ImageJ	Version 2.9.0
BDFacs Diva Software	Version 9.0.1
FlowJo	Version 10.8.2
MATLAB	2018A
RStudio	R 4.2.1/RStudio 2023.03.0

Abbreviations: Western Blot (WB), Flow Cytometry (FC), ImmunoFluorescence (IF)

Supplementary Table 2: Data summary

Figure 1 - Pathway activation
n=5, N=5

Fig1A - Ratio pMAPK/MAPK		
Condition	Ratio	SEM
Control	0.9722	0.2200
VEGF 5min	1.0264	0.0628
VEGF 15min	1.0816	0.1823
VEGF 30min	0.9328	0.2075

Fig 1B - Ratio pPLCɣ/PLCɣ		
Condition	Ratio	SEM
Control	0.9549	0.1446
VEGF 5min	0.8736	0.2270
VEGF 15min	1.0979	0.3102
VEGF 30min	0.5051	0.0269

Fig 1C - Ratio pPI3K/Actin		
Condition	Ratio	SEM
Control	1.0309	0.4738
VEGF 5min	1.2879	0.4734
VEGF 15min	1.2296	0.0487
VEGF 30min	1.2850	0.4305

Fig 1D - Ratio pSrc/Src		
Condition	Ratio	SEM
Control	0.8066	0.1298
VEGF 5min	1.1587	0.0691
VEGF 15min	1.2246	0.1022
VEGF 30min	0.9457	0.1157

Figure 2 - Aβ phagocytosis in vitro

Fig 2B - Aβo		*n=8, N=8*
Condition	FAM-Aβ MFI	SEM
Control 0h	1	0
Control 1h	32.6403	4.8169
Control 2h	53.8849	7.1657
Control 4h	57.9032	4.6917
Control 8h	39.48512	4.6838
VEGF 0h	1.07726	0.0418
VEGF 1h	35.2115	4.3932
VEGF 2h	58.7494	7.1824
VEGF 4h	68.3066	5.8063
VEGF 8h	46.9298	6.5324

Fig 2F - Aβf		*n=6, N=2*
Condition	FAM-Aβ MFI	SEM
Control 0h	1	0
Control 4h	4.275	0.3958
VEGF 4h	4.429	0.4711

Fig 2F - Aβf		*n=6. N=2*
Condition	%Phagocytic Cells	SEM
Control 0h	0.1	0
Control 4h	36.4833	1.2963
VEGF 4h	37.5833	2.3197

Fig 2D - Aβo degradation		*n=4, N=4*
Condition	FAM-Aβ MFI	SEM
Control 0h Degradation	1	0
Control 4h Degradation	0.6993	0.0896
Control 8h Degradation	0.3127	0.0373
VEGF 0h Degradation	0.9426	0.0557
VEGF 4h Degradation	0.7002	0.0582
VEGF 8h Degradation	0.3152	0.0417

Figure 3 - Aβ phagocytosis ex vivo
n=5, N=3

Condition	Median	1st Quantile	3rd Quantile	Mean	SEM
Control - No Cells	5.19	4.44	6.29	5.44	0.01
Control - Cells	5.00	4.30	6.09	5.31	0.01
VEGF - No Cells	5.25	4.44	6.40	5.49	0.011
VEGF - Cells	5.13	4.44	6.14	5.35	0.011

Fig 4 - ADAM cleavage activity

Fig 4B - Short stimulation		*n=15, N=5*
Condition	Mean Slope	SEM
Control	0.0021	0.0001
VEGF 0min	0.0027	0.0002
VEGF 15min	0.0026	0.0002
VEGF 30min	0.0022	0.0001

Fig 4C - Long stimulation		*n=15, N=5*
Condition	Mean	SEM
Control	100	3.7869
VEGF20h	64.7991	6.8584
VEGF6h	19.4079	11.5783
VEGF2h	40.0611	10.1301

Fig 5 - sTREM2

Fig 5A - ELISA sTREM2		*n=7-10, N=4*
Condition	Normalised Mean	SEM
Control	1	0.0772
VEGF-1h	1.5933	0.2536
VEGF-2h	1.2212	0.2139
VEGF-4h	0.9107	0.2019

Fig 5D - Aβo sTREM2		*n=8, N=3*
Condition	%Phagocytic cells	SEM
Control 4h	1	0.0163
sTREM2 4h	1.6091	0.1363

Fig 5C - Aβo sTREM2		*n=8, N=3*
Condition	FAM-Aβo MFI	SEM
Control 4h	1	0.0078
sTREM2 4h	1.2386	0.0435

Supplemental Figure 3 - Cryostat model checking

Sup Fig 3A		*n=10, N=3*
Condition	Aβ Intensity	SEM
No Cells	348	17.5
Cells	295	24.7

Sup Fig 3B		*n=10, N=3*
Condition	Relative Aβ area	SEM
No Cells	0.0719	0.005
Cells	0.0582	0.005

Sup Fig 3C		n=10, N=3
Condition	Mean Plaque Area	SEM
No Cells	510	16
Cells	420	17.9

Sup Fig 3E	*41 ROI, n=5, N=3*	
Density	Aβ area change	
Category 1	0.00896	0.006
Category 2	0.0166	0.005
Category 3	0.0121	0.009

Supplementary Table 3: Statistics summary

Figure 1 - Pathway Activation

Fig1A - pMAPK/MAPK
Kruskal-Wallis pv= 0.7271

Fig 1B- pPI3K/Actin
Kruskal-Wallis pv= 0.9529

Fig1C - pPLCɣ/PLCɣ	
Kruskal-Wallis pv= 0.04496	
Dunn post-hoc, Holm correction	p-value
Control vs VEGF 5 min	0.7307
Control vs VEGF 15min	0.3946
Control vs VEGF 30min	0.0309
VEGF 5 min vs VEGF 15min	0.6689
VEGF 5 min vs VEGF 30min	0.1227
VEGF 15 min vs VEGF 30min	0.0538

Fig 1D - pSrc/Src	
ANOVA pv= 0.04886	
Dunnet post-hoc	p-value
Control vs VEGF 5min	0.0819
Control vs VEGF 15min	0.0350
Control vs VEGF 30min	0.6858

Figure 2 - Aβ phagocytosis in vitro

Fig 2B - Aβo			
Mixed-effect model: lmer(MFI Normalized ~ Condition + Time + (1|Experiment), data)			
Factor	Beta	95% CI	p-value
Condition	6.32	[0.08, 12.56]	0.047
Time1h vs Time2h	21.76	[12.68, 30.83]	< 0.001
Time1h vs Time4h	28.54	[19.47, 37.61]	< 0.001
Time1h vs Time8h	8.65	[-0.43, 17.72]	0.061

Fig2D - Aβo degradation		
Linear model: aov(MFI Normalized ~ Condition + Time, data)		
ANOVA		
Factor	F-value	p-value
Condition	0.00008	0.9775
Time	44.3059	1.6E-05

Fig2F-2G - Aβf	
Wilcoxon test	p-value
Aβf MFI	0.9372
%Phagocytic	0.6991

Figure 3 - Aβ phagocytosis ex vivo

Mixed-effect model: lmer(logSurface~Condition*Cells+(1|Slice), data)			
Factor	Beta	95% CI	p-value
Condition	0.06	[-0.01, 0.13]	0.109
Cells	-0.13	[-0.16, -0.11]	< 0.001
Condition*Cells	-0.02	[-0.06, -0.02]	0.415

Fig 4 - ADAM cleavage activity

Fig 4B - Short stimulation	
Kruskal-Wallis pv= 0.00141	
Dunn post-hoc, Holm correction	
Comparison	p-value
Control vs VEGF0min	0.0063
Control vs VEGF15min	0.0123
Control vs VEGF30min	0.3929
VEGF0min vs VEGF15min	0.7380
VEGF0min vs VEGF30min	0.0127
VEGF15min vs VEGF30min	0.0204

Fig 4C - Long stimulation	
Kruskal-Wallis pv= 6.34e-07	
Dunn post-hoc, Holm correction	
Comparison	p-value
Control vs VEGF20h	0.0089
Control vs VEGF6h	0.0000
Control vs VEGF2h	0.0001
VEGF20h vs VEGF6h	0.0198
VEGF20h vs VEGF2h	0.1175
VEGF6h vs VEGF2h	0.1294

Fig 5 - sTREM2

Fig 5A - ELISA sTREM2			
Mixed-effect model: lmer(Normalised ~ Condition+(1|Experiment), data)			
Factor	Beta	95% CI	p-value
Condition			0.0321
Control vs VEGF1h	0.6	[0.15, 1.05]	0.011
Control vs VEGF2h	0.23	[-0.22, 0.68]	0.309
Control vs VEGF4h	-0.08	[0.53, 0.37]	0.710

Fig 5C - Aβo sTREM2
Wilcoxon test pv= 0.00016

Fig 5D - Aβo sTREM2
Wilcoxon test pv= 0.00016

Supplemental Figure 3 - Cryostat model checking

Sup Fig 3A
Wilcoxon test pv= 0.05243

Sup Fig 3B
Wilcoxon test pv= 0.04326

Sup Fig 3C
Wilcoxon test pv= 0.00151

Sup Fig 3E			
Mixed-effect model: lmer(AbetaChange ~ Density + (1|Slice) + (1|ROI), data)			
Factor	Beta	95% CI	p-value
Density Category 1	9.17e-03	[-2.69e-03, 0.02]	0.126
Density Category 2	7.18e-03	[-8.13e-03, 0.02]	0.348
Density Category 3	2.56e-03	[-0.02, 0.02]	0.798
